# Temporal Change in Blood Group after Bone Marrow Transplant: A Case of Successful ABO-Incompatible Deceased Donor Transplant

**DOI:** 10.1155/2020/7461052

**Published:** 2020-07-24

**Authors:** Susanna Lam, Sebastian Hultin, John Preston, Scott Campbell

**Affiliations:** ^1^Department of Renal Transplantation, Division of Surgery, Princess Alexandra Hospital, Brisbane, Australia; ^2^Queensland Kidney Transplant Service, Princess Alexandra Hospital, Brisbane, Australia; ^3^Department of Nephrology, Division of Medicine, Princess Alexandra Hospital at University of Queensland, Brisbane, Australia; ^4^Department of Urology, Division of Surgery, Princess Alexandra Hospital, Brisbane, Australia

## Abstract

ABO-incompatible kidney transplantation has been successfully utilised in a deceased donor and living donor kidney transplantation to improve organ utilisation and decrease waiting times. We describe a case of a successful, unanticipated ABO-incompatible donation after cardiac death (DCD) kidney transplant in a patient who had a previous ABOi haematopoietic stem cell transplant (HSCT) and had reverted to his original blood group B, after matching as a blood group A recipient with a blood group A donor. The recipient was unsensitized with a cPRA which was 0% and no donor-specific antibodies and zero HLA mismatch. An urgent anti-A titre was 1 : 2. Given the low antibody titres, we proceeded to transplantation. The patient developed delayed graft function and required dialysis on postoperative day 1 and day 2. The creatinine fell spontaneously on day 5, with progressively increased urine output and stable graft function on discharge at day 6. Anti-A titres were 1 : 1 on serial postoperative measurements. There were no rejection episodes, and the patient has a functioning graft at 16 months posttransplant. We describe a rare case in which the blood group can change after stem cell transplant and should be checked. We also demonstrate that a DCD ABOi transplant in the context of low anti-A titres for a patient with previous ABOi stem cell transplant can be performed successfully with standard immunosuppression.

## 1. Introduction

We describe a case of a successful, unanticipated ABO-incompatible deceased donor kidney transplant, following a change in blood group in a patient with a previous bidirectional ABO-incompatible haematopoietic stem cell transplant (HSCT). This patient was blood group A at the time of work-up and wait listing and was subsequently matched to a blood group A deceased donor kidney. At time of transplant, however, repeat protocol blood group testing revealed the recipient was blood group B. As low anti-A titre levels were detected, an unanticipated ABO-incompatible transplant was performed, with a standard immunosuppressive protocol and without plasmapheresis.

ABO-incompatible kidney transplantation has been successfully performed in living donor recipients with comparable outcomes to ABO-compatible transplants [1]. This usually requires a desensitization regimen of plasma exchange and immunoadsorption columns to reduce the antiblood group antibody titres, and in the past, splenectomy was performed to allow for immunologic accommodation [2]. However, ABO-incompatible transplants have been successful without the need for antibody removal, using standard immunosuppression in the context of low antiblood group antibodies [3].

ABO-incompatible kidney transplantation in deceased donors has been explored more recently in a bid to increase the utility of scarce resources for organs which may otherwise be discarded, as well as decrease organ discard and reduce the wait times for patients who would otherwise have a long wait time [4–6]. Notably, this has occurred in the setting of blood group A2 or A2B donors being transplanted into blood group type B or O with low anti-A haemagglutinin titres without the need for a desensitization regimen [4, 7, 8]. More recently, ABOi transplants from a deceased donor with blood group A1B donors have been utilised in patients with low antiblood group antibody titres in order to reduce the discard rate of AB blood group donors [5].

HSCT across the ABO barrier is not uncommon [9]. Five percent are bidirectionally incompatible, whereby the recipient develops the red blood cell type of the donor, and there are antibodies against both donor and recipient ABO blood group antigens, such as a blood group A donor to a blood group B recipient [10, 11]. Although often clinically irrelevant, relapse can occur in a bidirectionally incompatible HSCT recipient, which leads to reversion to the original ABO blood type [12, 13]. Relapse rates post ABOi HSCT are conflicting and likely dependent on other additional factors [11, 14, 15]. In the context of solid organ transplantation, potential blood group conversion and/or disease relapse needs to be an important consideration in the work-up of a recipient. This is particularly relevant for patients on a deceased donor transplant wait list, with potentially prolonged waiting times. A bidirectional incompatible HSCT patient with a failing graft may result in unanticipated ABO incompatibility at the time of transplant, as described in this case report.

## 2. Consent

Informed written consent was obtained from the patient for the use of their clinical information in the publication of this case report.

## 3. Case Description

This patient was a 66-year-old male, with a BMI of 31 kg/m^2^, listed for deceased donor transplantation due to end-stage kidney disease (ESKD) secondary to membranous nephropathy. He commenced haemodialysis in 2018 and had a residual urine output of 500 mls per day. The patient received a curative allogenic bidirectionally incompatible stem cell transplant from his brother in 2007 (donor group A; recipient group B) for myelofibrosis. He also had a splenectomy for essential thrombocytosis and massive splenomegaly. His other comorbidities include type 2 diabetes mellitus, hypertension, dyslipidemia, paroxysmal atrial fibrillation, and x-linked ichthyosis.

Prior to listing for deceased kidney transplantation, the patient had a recurrence of myelofibrosis that did not respond to interferon or donor lymphocyte infusion. His haematological prognosis, however, was deemed to be reasonable (estimated minimum 80 months) with a bone marrow biopsy prior to transplant listing that showed a hypercellular bone marrow with patchy fibrosis without leukemic transformation. He was listed for kidney transplantation in January 2018. His pretransplant work-up was unremarkable with immunotyping confirming blood group A and mild sensitization (peak cPRA 12% attributed to HSCT and associated blood transfusions). His calculated PRA (cPRA) at time of transplant was 0%. He had normal cardiovascular assessment. He had a transthoracic echocardiogram showing normal left and right ventricular size and function without valve pathology and with estimated left ventricular ejection fraction (LVEF) of 75%, and a stress myocardial perfusion scan revealed no clinically significant abnormality. He had suitable anatomy for transplantation with a normal aortoiliac duplex scan; however, he did have central obesity.

In February 2019, after 1 year on the transplant waiting list, the patient was offered a donor kidney after circulatory death (DCD). The kidney was blood group A and HLA identical between the donor and recipient (HLA A1,1; A2,2; B7,7; DR 1515; DQ6,6), without donor-specific antibodies. The T cell and B cell CDC crossmatches were negative. The donor was CMV negative and EBV positive; the recipient was CMV positive and EBV positive.

Admission blood showed a haemoglobin level of 101 g/L and white cell count of 8.6 × 10^9^/L corrected for promyelocytes. His biochemistry showed hyperkalemia (serum potassium 7.1 mmol/L) requiring preoperative dialysis with the remainder of his biochemical parameters being unremarkable. Routine blood group analysis, however, showed he was blood group B. Repeat redrawn blood testing confirmed blood group B. An urgent anti-A titre was performed and was 1 : 2. Due to excellent allelic matching and good kidney quality, the decision was made to proceed with an ABOi transplantation protocol.

The patient received standard immunosuppression regimen at our institution for standard immune risk patients (Basiliximab 20 mg at induction and on day 4, methylprednisolone 500 mg at induction and 12 hours post crossclamp release). The kidney transplant was performed in the right iliac fossa as previously described [16].The warm ischaemic time was 45 minutes and cold ischaemic time was 17 hours. He was maintained on prednisolone, myfortic 720 mg twice daily, and tacrolimus with a target trough level of 8-10 *μ*g/L. Standard prophylactic medications included piperacillin-tazobactam at induction, Bactrim® 400/80 mg daily, valganciclovir 450 mg adjusted to renal function, and amoxicillin 250 mg due to previous splenectomy.

His postoperative course was complicated by an episode of atrial fibrillation with rapid ventricular response, steroid-induced hyperglycaemia, and delayed graft function requiring haemodialysis on day 1 due to hyperkalemia (serum potassium 6.1 mmol/L). He required a blood transfusion of 1 unit of blood for a haemoglobin level of 69 g/L on day 2 post without stigmata of bleeding. His creatinine fell spontaneously on day 5, with progressively increasing urine output. No protocol biopsies were undertaken nor were they clinically indicated for this patient. Anti-A titres were 1 : 1 on day 1 with repeat titres consistently measured 1 : 1 postoperatively and at follow-up. At the time of last follow-up, 16 months posttransplant, he remains well with a stable renal function with creatinine of 135 *μ*moles and eGFR of 52 mL/min/1.73 m^2^.

## 4. Discussion

This is the first described case of unexpected ABO-incompatible solid organ transplantation due to blood group class switching following bidirectional ABOi HSCT failure due to myelofibrosis recurrence. Although successful kidney transplantation after haematopoietic stem cell transplantation has been described [17, 18], these cases were for living donor kidney transplants from their HLA identical or haploidentical donor or from different deceased donors [17, 19–21]. These were also in patients with ABO-compatible blood groups.

ABO-incompatible allogenic haematopoietic stem cell transplants (HSCT) have been used in the management of the haematologic malignancies [11]. In major, minor, or bidirectional ABO incompatibility, ABO grouping challenges post engraftment can occur. In the case of bidirectional incompatibility, as engraftment occurs, the donor red blood cells are detected and antibodies against the donor disappear [10, 11, 13]. Recommendations for blood transfusion support are dependent on the phase of HSCT; once blood group switching is confirmed, blood transfusions are generally of donor type [11–13]. In this case, the recipient had a HSCT 12 years earlier and was confirmed to be blood group A until at least June 2018, when he underwent routine annual HLA tissue typing and antibody testing. The patient received a kidney offer from a DCD donor (blood group A) and on perioperative blood testing was unexpectedly noted to be blood group B, the patient's pre-SCT blood type. This was confirmed again on a repeat blood group.

It is expected that after full engraftment, the recipients' ABO “forward type” should convert to that of the donor. However, there have been cases of patients who have undergone ABOi-incompatible HSCT who retained their original blood group, despite molecular evidence of full donor engraftment [12]. Other studies have shown that there may be a persistence of weak AB antigen on the patient's red blood cells that may affect the choice of ABO group and subsequently the provision of blood components and transfusions [22]. Furthermore, recurrence of his original haematological disease may have favoured his original blood type expression and development of a chimeric bone marrow.

Nevertheless, in this case, we were able to test for anti-A antibody levels in the recipient, and because the titres were low (1 : 2), we proceeded with an ABO-incompatible transplant with standard immunosuppression and without the need for desensitization regimen. ABOi haemagglutinin titre desensitization regimens are centre dependent and, if titres are low, may require only standard immunosuppression [3, 23].

ABOi deceased donor kidney transplantation is not common in Australia. The allocation process for deceased donor kidney involves allocation based on blood group-compatible or blood group-acceptable transplants. In exceptional circumstances, such as in a rare blood group where there is no recipient on the wait list with a compatible blood group and HLA compatible, then an ABOi transplant may be considered [24]. ABOi deceased donor transplantation for A1B donors into patients with blood groups A and B, where there were no suitable AB recipients, has been described [5]. Other reports of unintentional ABO-incompatible kidney transplants have occurred in the context of blood group A1B donors into A2B recipients, with low anti-A1 antibody titres [25, 26] or no anti-A titres without the need for a pretransplant desensitization regimen [27].

This case highlights the need for ongoing vigilance for patients who have had prior ABOi stem cell transplants, particularly in identifying the risk for potential of regrafting or change of blood group type. It further highlights the need for a stringent perioperative checklist such as the World Health Organization surgical checklist [28] but more specifically a focus in transplant surgery, particularly with checking blood grouping and immunosuppression requirements to reduce morbidity and mortality. Furthermore, for any patient with a history of bone marrow transplantation, blood grouping needs to be checked at the time of surgery and not assumed to be stable over time.

## 5. Conclusion

We demonstrate a successful DCD ABOi transplant with standard immunosuppression in a recipient with possible bone marrow chimerism. Despite confirmed ABO conversion after stem cell transplantation, the recipient's original blood group B cells had regrafted with only low anti-A titres. Therefore, blood grouping may not be static and, although a rare circumstance, in the setting of bone marrow transplantation, can revert unexpectedly to the previous blood group and must be confirmed prior to transplantation.
